# Thai Fermented Soybean (Thua-Nao) Prevents Early Stages of Colorectal Carcinogenesis Induced by Diethylnitrosamine and 1,2-Dimethylhydrazine Through Modulations of Cell Proliferation and Gut Microbiota in Rats

**DOI:** 10.3390/nu16203506

**Published:** 2024-10-16

**Authors:** Sirinya Taya, Sivamoke Dissook, Jetsada Ruangsuriya, Supachai Yodkeeree, Kongsak Boonyapranai, Teera Chewonarin, Rawiwan Wongpoomchai

**Affiliations:** 1 Functional Food Research Unit, Multidisciplinary Research Institute, Chiang Mai University, Chiang Mai 50200, Thailand; 2 Department of Biochemistry, Faculty of Medicine, Chiang Mai University, Chiang Mai 50200, Thailand; 3 Research Institute for Health Sciences, Chiang Mai University, Chiang Mai 50200, Thailand

**Keywords:** Thai fermented soybean, Thua-nao, aberrant crypt foci, cell proliferation, gut microbiota

## Abstract

Background: Thua-nao is a traditional fermented soybean product widely consumed in the northern areas of Thailand. There has been little research on the biological activity of Thua-nao, particularly its anticancer properties. Objectives: The objective of this study was to examine the cancer chemopreventive effects of dried Thua-nao on liver and colorectal carcinogenesis induced by carcinogens in rats. Methods: Rats were injected with diethylnitrosamine (DEN) and 1,2-dimethylhydrazine (DMH) to induce preneoplastic lesions. Rats orally received dried Thua-nao for 13 weeks. The preneoplastic lesions, including glutathione *S*-transferase placental form (GST-P)-positive foci and aberrant crypt foci (ACF), were evaluated in the liver and colon, respectively. The cancer chemopreventive mechanisms of dried Thua-nao on liver and colorectal carcinogenesis were examined. Results: Dried Thua-nao administration suppressed colorectal aberrant crypt foci. Moreover, dried Thua-nao reduced proliferation cell nuclear antigen (PCNA)-positive cells in the colon. Interestingly, dried Thua-nao modulated the gut microbiota in DEN- and DMH-induced rats. Isoflavones, including genistein and daidzein, represent promising chemopreventive agents in dried Thua-nao. Conclusions: In conclusion, these results highlight the cancer chemopreventive effect of dried Thua-nao in DEN and DMH-induced colorectal carcinogenesis through cell proliferation reduction and gut microbiota modulation.

## 1. Introduction

Cancer is a serious global public health issue, with colon and liver cancers being common and destructive [1]. Colon cancer, or colorectal cancer (CRC), ranks as the third most prevalent cancer globally and is the second leading cause of cancer-related mortality worldwide [1]. A sedentary lifestyle, obesity, smoking, and excessive alcohol consumption are among the lifestyle factors that can influence colorectal cancer [2]. Primary prevention strategies, including the implementation of early detection through screening, the avoidance of risk factors, and the maintenance of a healthy lifestyle, can significantly reduce the incidence and impact of colorectal cancer [2]. Liver cancer, or hepatocellular carcinoma (HCC), ranks as the sixth most prevalent cancer globally and is the third primary cause of cancer-related mortality worldwide [1]. Common risk factors for liver cancer are frequently associated with chronic liver disease, including hepatitis B or C infection, cirrhosis, or environmental factors such as heavy alcohol intake, smoking, and exposure to aflatoxin [3]. Both colorectal cancer and liver cancer have common risk factors, including lifestyle decisions such as diet, obesity, and alcohol intake. Particularly, diet is one of the environmental factors that is associated with an increased risk of cancer. However, dietary patterns that are appropriate may mitigate the likelihood of developing cancer [4,5]. The American Cancer Society (ACS) provides dietary and physical activity recommendations for preventing the progression of cancer [6]. These recommendations include the consumption of foods that are rich in nutrients, vegetables, legumes, fruits, and whole grains [6]. Furthermore, several studies have indicated that consumption of vegetables, unrefined grains, and fermented foods is associated with a reduced risk of cancer; naturally occurring chemicals in these foods act as chemopreventive agents against cancer [7,8,9].

Currently, researchers are investigating the influence of microbial community alterations on cancer. These microbial communities play important roles in several types of cancers, including breast, liver, and colorectal cancer. Tens of thousands of microorganisms live in the human digestive system, including bacteria, archaea, fungi, protozoa, and viruses, with bacteria making up the vast majority [10]. Gut dysbiosis is a condition characterized by changes in the composition and function of the gut microbiota due to an imbalance between beneficial and harmful microorganisms. Dysbiosis can be classified into three categories: depletion of helpful microorganisms, proliferation of harmful microorganisms, and reduction in microbial variety [11]. Dysbiosis is a contributing factor to various clinical illnesses, especially cancers [11]. Nutrition profoundly affects the composition of the human gut microbiota. For instance, a diet abundant in high-fermented foods enhances the diversity of bacteria in the body while concurrently reducing inflammatory indicators [12].

Thua-nao is a traditional fermented soybean product that is commonly consumed in the northern regions of Thailand, including Chiang Mai, Chiang Rai, Lampang, and Mae Hong Son. In addition, there are similar fermented soybean products in many countries, including natto in Japan, kinema in India, and chongkukjang in Korea [13]. The traditional preparation of Thua-nao involves immersing the soybeans overnight and boiling them for 4 h until they become completely soft. Subsequently, the cooked soybeans are naturally fermented in bamboo baskets lined with banana leaves for a few days. Although fresh Thua-nao can be consumed after fermentation, most people prefer steaming or grilling before consumption. Cooked Thua-nao can be shaped into a flat disk and sun-dried to create a variety form known as Thua-nao kab. The dried product can be mashed into a fine powder and stored for several months [13]. The previous study found that the proximate analysis of commercial Thua-nao collected from six local markets in Chiang Mai comprised 38.94–42.06% crude protein, 20.37–25.22% fat, 12.92–28.06% crude fiber, 4.70–5.44% ash, and 57.22–64.78% moisture [14]. Thua-nao is naturally fermented by indigenous microorganisms, especially the *Bacillus* spp. group [15,16]. Pakwan and colleagues reported that *Bacillus* was the most common bacteria in Thua-nao, while Lactic acid bacteria (LAB) of the family *Leuconostocaceae* were also present in substantial quantities [15]. Consequently, Thua-nao is a nutrient-rich source of beneficial bacteria that has the potential to be a probiotic food [15]. Furthermore, Thua-nao is abundant in bioactive compounds, including isoflavones and short-chain fatty acids; it also possesses biological activities, such as antioxidant activity, which are beneficial for health [14,16]. Nonetheless, the exact health benefits of Thua-nao consumption are still unclear, and the chemopreventive efficacy of Thua-nao against cancer in animal models remains inadequately explored. Diethylnitrosamine (DEN) and 1,2-dimethylhydrazine (DMH) are recognized as carcinogens for the liver and colon, respectively, and both undergo metabolism by cytochrome P450 in the dual organ carcinogenicity assay [17]. In line with the three Rs of animal research, these carcinogens were given to the same rats in this investigation to minimize the number of animals involved in the experimental process [17,18,19]. Therefore, to elucidate the cancer chemopreventive properties of Thua-nao, a model of liver and colon carcinogenesis induced by DEN and DMH in rats was employed to examine its effects on the early stages of liver and colorectal carcinogenesis in rats, as well as its potential inhibitory mechanisms.

## 2. Materials and Methods

### 2.1. Preparation of Thua-Nao

Soybeans were purchased from a commercial brand in Thailand. After being soaked overnight, soybeans were boiled for three hours and naturally fermented in an incubator at 37 °C for 3 days. The fresh Thua-nao was dried in a hot air oven at 50 °C for 48 h, subsequently being milled with a powder grinder. The dried powder was stored at −20 °C until use. The animal equivalent dose (AED) of dried Thua-nao was calculated based on a 75 kg Thai adult’s average daily Thua-nao intake, which is roughly 12 g (160 mg/kg bw/day). A conversion factor of 6.2 is utilized to multiply by the human dose in order to calculate AED based on the difference in body surface area between rats and humans [20]. Therefore, the equivalent human dose of dried Thua-nao (160 mg/kg bw/day) is equivalent to a dose of 992 mg/kg bw/day in rats. In the cancer chemopreventive study, the daily animal doses of dried Thua-nao were 100 and 1000 mg/kg bw. Additionally, the BAM method was used to count the *Escherichia coli* and coliform bacteria in the dried powder of Thua-nao in order to check the food safety in Thua-nao for pathogenic microorganism contamination [21].

### 2.2. Chemical Constituents of Dried Thua-Nao

Dried Thua-nao powder was soaked in 70% ethanol for two days and then extracted again. The filtrate was evaporated and freeze-dried to obtain an ethanolic extract. This extract was used to measure the contents of phenolic compounds and isoflavones.

The content of total phenolic compounds in the ethanolic extract was measured spectrophotometrically using the Folin–Ciocalteu method [22].

The isoflavone contents of the ethanolic extract were examined by high-performance liquid chromatography (HPLC). The analysis was performed using a reverse phase C18 column (Agilent 4.6 mm × 250 mm, 5 μm) and analyzed on an Agilent HPLC 1260 (Agilent Technologies, Santa Clara, CA, USA). Gradient elution was carried out using a mixture of 1% trifluoroacetic acid (TFA) in water and methanol. The flow rate was 1 mL/min, and the injected volume was 10 μL. The absorbances at 254 and 260 nm were monitored. The isoflavone contents were determined and quantified using calibration curves for daidzin, daidzein, genistin, genistein, glycitin, and glycitein.

### 2.3. Animal and Experimental Protocol

The Institutional Animal Care and Use Committee of the Faculty of Medicine, Chiang Mai University, approved the animal study protocol (no. 26/2565). The animal study protocol was performed in accordance with the relevant guidelines and regulations. This protocol follows the recommendations in the ARRIVE guidelines. Male Wistar rats (three-week-old rats) were acquired from Nomura Siam International Co., Ltd., Bangkok, Thailand. All rats were maintained under standard conditions at 25 °C with a 12 h light/dark cycle and were provided with a commercial basal diet (C.P. mice feed 082G, Samut Prakan, Thailand, Table S1) and water.

**Figure 1:**
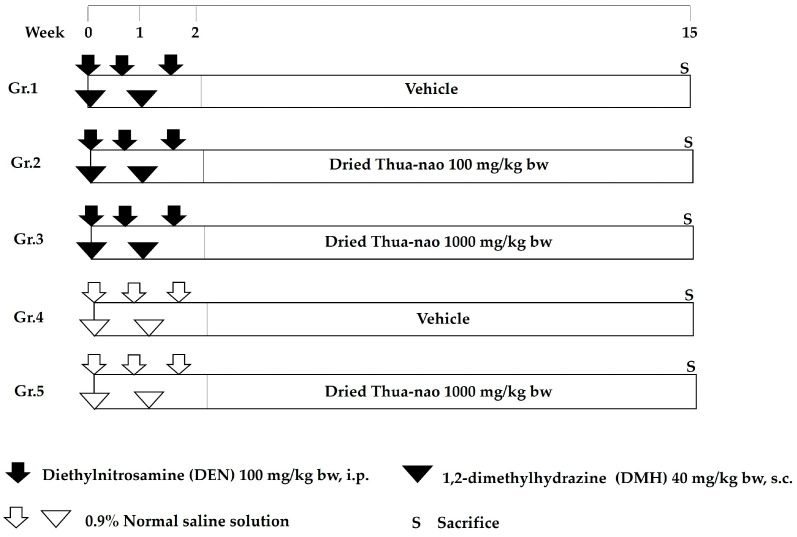
Animal experimental protocol for a cancer chemopreventive study of dried Thua-nao in DEN- and DMH-treated rats.

The animal experimental design is illustrated in Figure 1. All rats were randomly divided into five groups. Rats in groups 1 through 3 were injected with 100 mg/kg bw of DEN (i.p.) on days 0, 4, and 11 and 40 mg/kg bw of DMH (s.c.) on days 0 and 7, whereas rats in groups 4 and 5 were injected with 0.9% normal saline solution. Dried Thua-nao at 100 and 1000 mg/kg bw were oral gavage fed 5 days a week for 13 weeks, whereas distilled water was used as a vehicle. Throughout the experiment, body weight and intake of diet and water were recorded. At the end of the experiment, rats were euthanized with overdosed isoflurane. Blood was collected for aspartate aminotransferase (AST) and alanine aminotransferase (ALT) determinations at the Small Animal Hospital, Faculty of Veterinary Medicine, Chiang Mai University. Internal organs, including the liver, spleen, and kidneys, were collected and weighed. The liver was sectioned into three pieces and fixed in 10% buffered neutral formalin (BNF) for determinations of glutathione *S*-transferase placental form (GST-P)-positive foci and proliferation cell nuclear antigen (PCNA)-positive cells by immunohistochemistry. The colon was expanded with 10% BNF and placed on ice for 30 min. Then, the colon was divided into three segments, namely proximal, distal, and rectum segments, and flattened in 10% BNF for evaluation of aberrant crypt foci (ACF); cross-section paraffin-embedded colonic tissues were used for determining PCNA-positive cells by immunohistochemistry. Furthermore, feces were collected from the rat anus and kept at −80 °C for analysis of the composition of the fecal intestinal microbiota.

#### 2.3.1. Determination of Preneoplastic Lesions in DEN- and DMH-Treated Rats

The preneoplastic lesions in the liver were investigated by immunohistochemical staining with the anti-GST-P antibody (MBL, Nagoya, Japan), as previously described [23]. Briefly, liver sections of 4 µm thickness were deparaffinized and dehydrated. The liver sections were soaked in 3% hydrogen peroxide (H_2_O_2_) and 1% skimmed milk to prevent the activity of pseudoperoxidase and non-specific binding of proteins, respectively. Following incubation with a rabbit polyclonal anti-rat GST-P antibody, the liver sections were incubated with an anti-rabbit IgG biotinylated antibody. Subsequently, the liver sections were soaked with diaminobenzidine (DAB), while hematoxylin was used as a counterstain. The area and number of GST-P-positive foci exceeding 0.02 mm^2^ were analyzed. The results were represented as the area and number of GST-P-positive foci per liver area (cm^2^).

The ACF in the colon was evaluated using methylene blue staining. Methylene blue staining was applied to each segment’s flattened colon. The number and size of ACF were counted under the light microscope following Bird’s criteria [24,25]. The results were expressed as the size and number of ACF per rat.

#### 2.3.2. Determination of PCNA-Positive Cells by Immunohistochemistry

The immunohistochemistry of PCNA-positive cells was analyzed in the liver and colon as detailed by Chariyakornkul et al. [18]. The paraffin-embedded liver sections and colon cross-sections were deparaffinized. The sections were first incubated with a monoclonal mouse anti-rat PCNA antibody (BioLegend, San Diego, CA, USA), followed by biotinylated antibodies and an Elite avidin-biotin complex kit (Vector Laboratories, Inc., Burlingame, CA, USA). The PCNA-positive cells were counted using a light microscope.

#### 2.3.3. Investigation of the Fecal Gut Microbiota Composition in Rats

The gut bacterial profiles were analyzed using 16s rRNA amplicon sequencing. First, bacterial DNA was extracted from frozen feces using a QIAamp DNA microbiome kit according to the instructions provided in the user manual. The hypervariable V4 region of the 16S rRNA gene from gut microbiota was sequenced using primers 515F (5′-GTGCCAGCMGCCGCGGTAA-3′) and 806R (5′-GGACTACHVGGGTWTCTAAT-3′). Following PCR amplification, paired-end sequencing (2 × 250 bp) was performed on the Illumina NovaSeq 6000 platform, according to the manufacturer’s 16S genomic sequencing library preparation protocol. The raw sequencing data were processed to remove low-quality reads, merge overlapping reads, and identify and remove chimeric sequences. High-quality clean sequences were then annotated at various taxonomic levels (phylum to genus) using the Quantitative Insights Into Microbial Ecology 2 (QIIME2; version 2021.8.0). Taxonomy classification was performed against the SILVA 16S rRNA gene reference database release 138. The alpha diversity metrics, including species richness, were reflected by the Shannon index. Beta diversity was evaluated using the Unifrac phylogenetic distance. Data visualization was performed using R software version 4.3.2.

### 2.4. Statistical Analysis

All data are presented as mean ± SD values. The significance of differences between groups was assessed by one-way ANOVA, followed by Tukey’s multiple comparison test, utilizing GraphPad Prism 9.0 software (GraphPad Software, Boston, MA, USA). Statistical significance was considered as *p*-value < 0.05.

The Kruskal-Wallis test was applied to assess differences in alpha-diversity among the groups. For pairwise comparisons between groups, the Wilcoxon rank-sum test was used. Additionally, PERMANOVA was employed to evaluate statistical differences in beta-diversity across groups.

## 3. Results

### 3.1. Chemical Components and Microbial Contamination of Dried Thua-Nao

The chemical constituents of dried Thua-nao are shown in Table 1. One hundred grams of dried Thua-nao contained 1180.21 ± 26.63 mg of phenolic compounds, as measured by spectrophotometry. The major isoflavones in dried Thua-nao were daidzein and genistein. To consider food safety in terms of pathogenic microorganism contamination in Thua-nao, total coliform bacteria were determined primarily, and it was found that there were >1100 most probable number (MPN)/g of total coliform bacteria. However, fecal coliform bacteria and *E*. *coli* were 3.6 MPN/g and <3 MPN/g, respectively. It is concluded that the Thua-nao used in this study is safe from bacterial contamination based on the presence of fecal coliform bacteria and *E*. *coli* being less than the limits (fecal coliform bacteria and *E*. *coli* < 500 and 3 MPN/g, respectively).

### 3.2. Effect of Dried Thua-Nao on Preneoplastic Lesion Formation in the Liver and Colon of DEN- and DMH-Treated Rats

**Figure 2:**
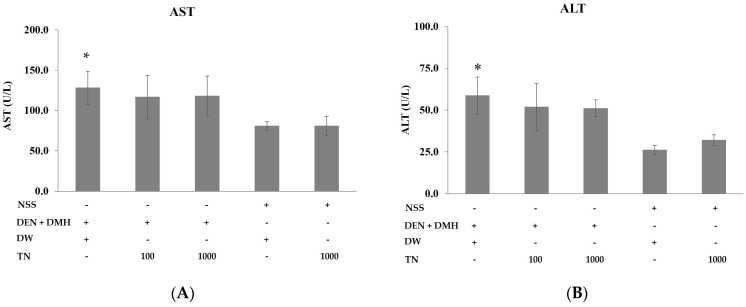
Effects of dried Thua-nao on serum liver function enzyme levels in rats. The levels of (**A**) aspartate aminotransferase (AST) and (**B**) alanine aminotransferase (ALT) in rats. Diethylnitrosamine (DEN); 1,2-dimethylhydrazine (DMH); normal saline solution (NSS); Thua-nao (TN); distilled water (DW). * Significantly different compared to the NSS-treated group (*p* < 0.05).

According to overall findings, the administration of dried Thua-nao to rats receiving NSS led to a notable rise in their general parameters, including final body weight and diet consumption, compared to rats treated with only NSS. The injections of DEN and DMH tended to reduce the final body weight of the rats, while administration of dried Thua-nao at a low dose tended to increase the final body weight and significantly increase diet intake when compared to DEN- and DMH-treated rats (Table 2). Furthermore, administering dried Thua-nao did not affect the absolute and relative kidney and spleen across the groups. Nevertheless, the administration of dried Thua-nao at a high dose to rats treated with NSS resulted in a significant increase in the absolute weight of the liver, although no significant difference was observed in the relative weight of the liver (Table 3). The administration of DEN and DMH induced liver damage in rats, as detected by increasing serum AST and ALT levels; however, feeding dried Thua-nao did not reduce serum AST and ALT when compared to DEN- and DMH-treated rats (Figure 2). Interestingly, feeding dried Thua-nao did not change levels of liver function enzyme when compared to NSS-treated rats, suggesting that dried Thua-nao is non-hepatotoxic to rats.

Dried Thua-nao neither induced GST-P-positive foci nor ACF in NSS-treated rats (Table 4), suggesting that dried Thua-nao is non-carcinogenic to rat liver and colon. Rats treated with DEN and DMH exhibited a marked elevation in GST-P-positive foci and ACF formation compared to NSS-treated rats. The administration of dried Thua-nao at a dose of 1000 mg/kg bw tended to reduce the number and size of GST-P-positive foci in DEN- and DMH-treated rats, but the results were not significantly different when compared with carcinogen-induced rats. Notably, the dried Thua-nao at a dose of 1000 mg/kg bw significantly decreased the number of ACF in the colon of DEN- and DMH-treated rats, while the size of the aberrant crypt did not change. The results suggest that dried Thua-nao could reduce the development of colorectal carcinogenesis in rats. Additionally, administering dried Thua-nao to rats treated with NSS did not cause the formation of GST-P-positive foci in the liver or ACF in the colon, indicating that dried Thua-nao does not have carcinogenic effects in the rat liver and colon.

### 3.3. Effect of Dried Thua-Nao on Cell Proliferation in Liver and Colon of DEN- and DMH-Treated Rats

**Figure 3:**
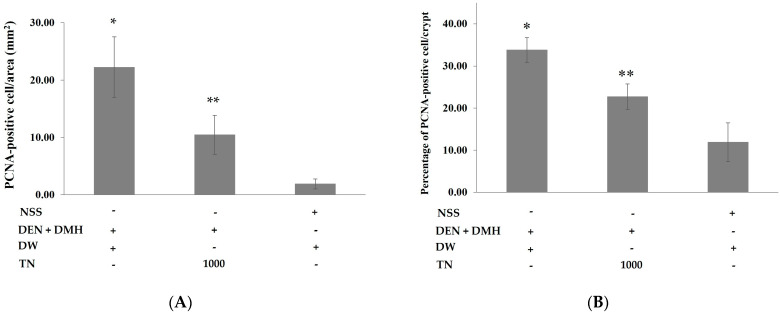
Effect of dried Thua-nao on cell proliferation in rat liver and colon tissues. PCNA-positive cells in the (**A**) liver and (**B**) colon were quantified by immunohistochemical staining. Diethylnitrosamine (DEN); 1,2-dimethylhydrazine (DMH); normal saline solution (NSS); Thua-nao (TN); distilled water (DW). * Significantly different compared to the NSS-treated group (*p* < 0.05). ** Significantly different compared to the DEN- and DMH-treated group (*p* < 0.05).

Following the administration of dried Thua-nao at a high dose, which demonstrated a decrease in preneoplastic lesions of carcinogen-treated rats, the mechanisms of cell proliferation were investigated. The number of PCNA-positive cells in the liver and colon of rats that were treated with DEN and DMH significantly increased when compared to rats that were treated with NSS. Administration of the dried Thua-nao at a high dose significantly reduced the number of PCNA-positive cells in both the liver and the colon when compared to DEN- and DMH-treated rats (Figure 3). Based on these findings, dried Thua-nao may prevent the formation of preneoplastic lesions by modulating cell proliferation in the liver and colon carcinogenesis.

### 3.4. Effect of Dried Thua-Nao on Bacterial Profile in Rats

In this study, the impact of Thua-nao on the gut microbiota of rats treated with DEN and DMH was investigated. Our in-depth 16S rRNA amplicon sequencing analysis showed that the gut microbiome’s composition and diversity changed significantly after Thua-nao administration.

**Figure 4:**
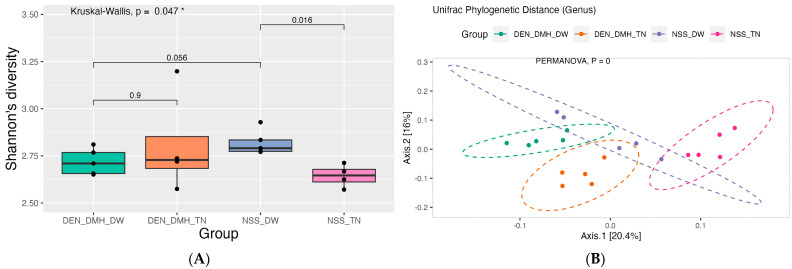
Alternation in bacterial diversity within each group. (**A**) Boxplot of gut microbial alpha-diversity based on Shannon index. (**B**) Beta-diversity of gut microbiota composition structure based on Unifrac phylogenetic distance. Diethylnitrosamine (DEN); 1,2-dimethylhydrazine (DMH); normal saline solution (NSS); Thua-nao (TN); distilled water (DW); *n* = 5 rats in each group. Statistical significance was determined using the Kruskal-Wallis test to assess differences among groups, with Wilcoxon rank-sum tests used to determine pairwise differences between groups. The level of statistical significance is denoted as * *p* < 0.05.

This study found that the alpha diversity metrics, particularly the Shannon index, showed a significant change in the Thua-nao administered to the NSS-treated rats (NSS_TN) compared to the NSS-treated rats (NSS_DW). These changes indicate a notable increase in microbial diversity, suggesting that consuming Thua-nao contributes to a more robust gut microbiota. This finding was depicted in Figure 4A, which shows a boxplot comparison of the alpha diversity between groups. Furthermore, beta-diversity analysis, utilizing Unifrac phylogenetic distance, demonstrated a clear separation between the groups (Figure 4B), signifying distinct gut bacterial communities in each group. Such differentiation underpins Thua-nao’s influential role in modulating the gut microbiome landscape, emphasizing its dietary significance.

**Figure 5:**
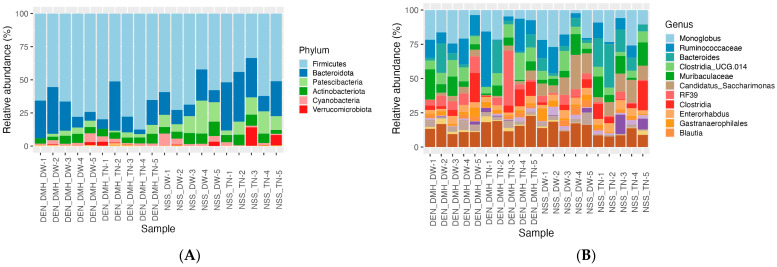
Percent relative abundances of the taxonomic compositions in each group at (**A**) the phylum and (**B**) genus levels. Diethylnitrosamine (DEN); 1,2-dimethylhydrazine (DMH); normal saline solution (NSS); Thua-nao (TN); distilled water (DW); *n* = 5 rats in each group.

The characterized bacterial composition at various taxonomic levels, from phyla to genera, is presented in Figure 5, revealing six bacterial phyla with differences in their relative abundance across the four groups (Figure 5A). Firmicutes were found to have a higher-level abundance in all of the groups, suggesting a foundational presence in the gut microbiome. At the genus level, a total of 155 microbial genera were annotated (Table S2), with the top 11 in abundance shown in Figure 5B, providing a clear visualization of the microbial distribution in each group.

**Figure 6:**
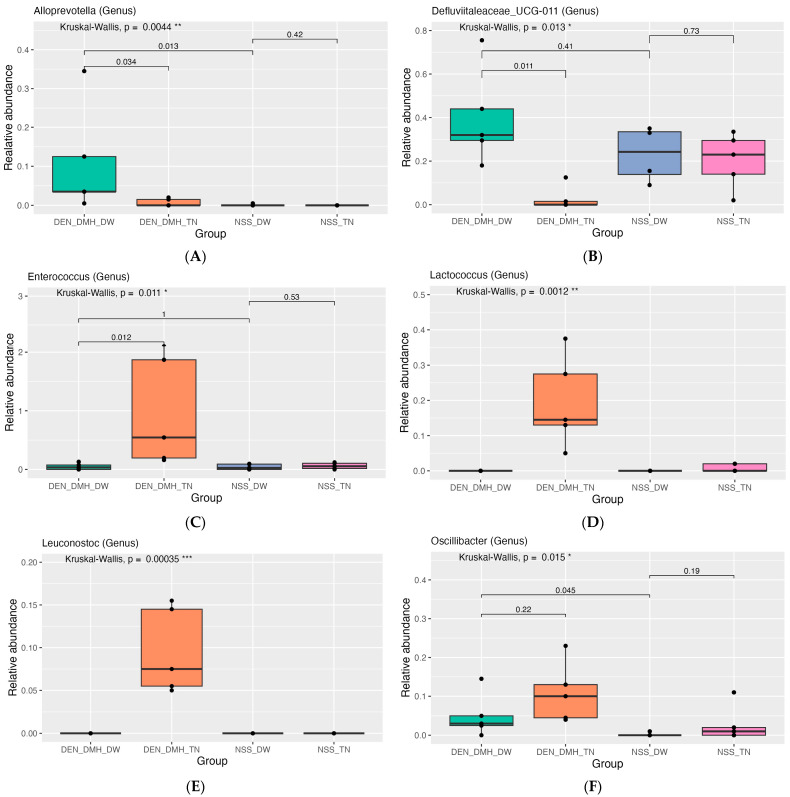

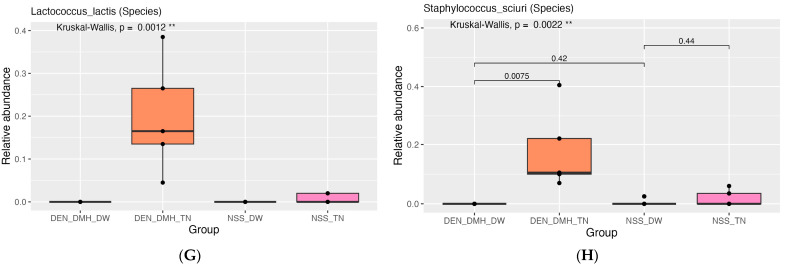
Relative abundance of the differential microbiota in each group. Relative abundance of (**A**) *Alloprevotella*, (**B**) *Defluviitaleaceae_UCG-011*, (**C**) *Enterococcus*, (**D**) *Lactococcus*, (**E**) *Leuconostoc*, (**F**) *Oscillibacter*, (**G**) *Lactococcus lactis*, and (**H**) *Staphylococcus sciuri*. Diethylnitrosamine (DEN); 1,2-dimethylhydrazine (DMH); normal saline solution (NSS); Thua-nao (TN); distilled water (DW); *n* = 5 rats in each group. Statistical significance was determined using the Kruskal-Wallis test to assess differences among groups, with Wilcoxon rank-sum tests used to determine pairwise differences between groups. Levels of statistical significance are denoted as * *p* < 0.05, ** *p* < 0.01, *** *p* < 0.001.

Furthermore, the relative abundance of the differential microbiota in each group is presented in Figure 6. Rats injected with DEN and DMH (DEN_DMH_DW) showed a marked increase in the abundance of *Alloprevotella* and *Oscillibacter* as compared to the NSS-treated group (NSS_DW). Administering dried Thua-nao to DEN- and DMH-treated rats (DEN_DMH_TN) significantly reduced the abundance of *Alloprevotella* and *Defluviitaleaceae_UCG-011*, along with an increase in the abundance of *Enterococcus*, *Lactococcus*, *Leuconostoc*, *Lactococcus lactis*, and *Staphylococcus sciuri* when compared to DEN- and DMH-treated rats (DEN_DMH_DW).

## 4. Discussion

Fermented soybean products are frequently employed as seasoning agents or condiments for enhancing the taste of food. Various fermented soybean products originating from diverse regions of Asia include tempeh, natto, miso, soy sauce, doenjang, and Thua-nao [13,26,27]. Fermented soybean products exhibit various bioactive compounds and potential health benefits, including antidiabetic, antioxidant, anti-inflammatory, antihyperlipidemic, and anticancer activities [27,28]. Thua-nao is a traditional fermented soybean product that is widely consumed in northern Thailand. However, there have been few studies on Thua-nao’s biological activities, particularly anticancer activity. The present study reveals the cancer chemopreventive effects of Thua-nao in a rat model of liver and colon carcinogenesis induced by DEN and DMH. DEN is commonly employed as a hepatocarcinogen in the process of chemically induced hepatocarcinogenesis, whereas DMH is frequently utilized as a colon carcinogen in animal models of chemically induced colon carcinogenesis. The dual organ carcinogenicity model, employing DEN and DMH as initiators, was utilized to induce the early stages of liver and colon carcinogenesis in rats [17]. GST-P is highly expressed during hepatocarcinogenesis in rats and has been used as a reliable marker for experimental hepatocarcinogenesis in rats [29]. In addition, ACF can be used as a reliable marker for chemically induced colorectal carcinogenesis in rats [30]. This model served as a valuable tool for investigating potential cancer chemopreventive agents [18,19].

Dried Thua-nao consumption at a high dose significantly increased both the final body and the liver weights, but the relative organ weight showed no statistical difference. The normalization of organ weights to the body is one of the procedures to reduce variations among groups in which their body weights are varied and finally presented in organ-to-body weight ratios [31]. In addition, those ratios are considered critical parameters for drug and substance toxicity determinations. Consequently, when the relative liver weight shows no statistical difference, it can be inferred that administering dried Thua-nao had no effect on the liver. 

Co-administration of DEN and DMH induced the formation of GST-P-positive foci in the liver and ACF in the colon. Thua-nao, however, did not inhibit GST-P-positive foci in DEN- and DMH-treated rats. Interestingly, Thua-nao reduced the total number of ACF but had no effect on the size of ACF in the colons of carcinogen-treated rats. Furthermore, the inhibitory effect of dried Thua-nao was observed only when administered in high doses, which were equal to human daily consumption. This underlines the role of Thua-nao in regulating the formation of preneoplastic lesions in the colon following carcinogen exposure in rats. In DMH-induced ACF development in rats, liver CYP2E1 catalyzes the biotransformation of DMH to methylazoxymethanol (MAM), which interacts with DNA, causing adduction at O^6^-methylguanine (O^6^-MeG), ultimately resulting in mutations and tumors [32]. In colonic mucosa cells, MAM, an electrophilic methylating chemical, can methylate DNA at guanine bases [33]. The number of ACF is thought to be related to O^6^-MeG. Furthermore, data have been published that indicate a correlation between the decrease in O^6^-MeG in the colonic mucosa and the decrease in ACF, or adenocarcinoma, in the rat colon [34]. Reducing phase I enzyme activity is, therefore, an effective approach for preventing reactive intermediates from generating DNA adducts [35,36]. Moreover, the conjugated MAM is hydrolyzed by intestine bacterial β-glucuronidase, which releases free MAM [37]. After free MAM is reabsorbed into the colonic epithelial cell, DNA adduct and mutation occur [38]. As a result, one potential approach to preventing colorectal carcinogenesis is to reduce the activity of bacterial β-glucuronidase. Numerous studies indicated that *Ficus dubia* latex extract and purple rice extract suppressed DMH-induced colorectal carcinogenesis by modifying xenobiotic metabolism in the liver and colon, resulting in decreased DNA adduct levels and a reduction in ACF numbers during the initiation stage [36,39].

One of the most important factors in cancer development is an imbalance between cell growth and apoptosis [40]. The proliferating cellular nuclear antigen (PCNA) is a reliable biomarker for cell proliferation [41]. This finding reveals that the administration of Thua-nao at a high dose significantly reduced the number of PCNA-positive cells in the colon in carcinogen-treated rats. Furthermore, this study reveals that genistein and daidzein are the most abundant isoflavones found in dried Thua-nao. These isoflavone aglycones are converted from their isoflavone glycosides by β-glycosidase, which is produced by microorganisms during the fermentation process [27]. Furthermore, recent studies indicate that many traditional Asian fermented soy products, such as Japanese natto, Indian kinema, and Thai Thua-nao, have a much higher content of isoflavone aglycone compared to unfermented soybeans [42,43]. A systematic review and meta-analysis reported that soy isoflavone consumption was significantly associated with a reduced risk of colorectal cancer [44]. Several studies have discovered that genistein effectively inhibited the development of colon cancer caused by carcinogens in in vitro studies. Zhang et al. reported that genistein suppresses azoxymethane-induced WNT/β-catenin signaling, avoiding early colon neoplasia [45]. Moreover, Sekar et al. found that genistein reduced ACF in DMH-induced rats by activating nuclear factor-erythroid 2 related factor 2 (Nrf-2) and modulating the expression of proliferative markers, including PCNA [46]. In human colon cancer cells, genistein promotes G2/M cell cycle arrest and apoptosis through an ATM/p53-dependent mechanism [47]. Hence, the probable cancer chemopreventive compounds in Thua-nao would likely comprise isoflavones, including genistein and daidzein.

The gut microbiota has a crucial role in the initiation and promotion of various types of cancer, especially malignancies in the gastrointestinal tract. Bacteria can indeed initiate chronic inflammation of the gastric mucosa, leading to permanent alterations in intestinal epithelial cells and increasing the susceptibility of individuals to cancer [48,49]. Notably, the gut microbiota in colorectal cancer is linked to certain strains of *Bacteroides fragilis*, *Escherichia coli*, *Clostridium* spp., *Streptococcus gallolyticus*, *Fusobacterium nucleatum*, *Streptococcus bovis*, and *Enterococcus faecalis* [49,50,51]. However, the role of *E. faecalis* remains controversial [52]. *E. faecalis* is a member of the *Firmicutes* phylum and is occasionally employed as a probiotic product [53]. Nevertheless, under certain circumstances, *E. faecalis* can have pathogenic effects and can be detrimental to the development of colorectal cancer due to its capacity to disrupt the DNA of colonic epithelial cells [54]. In this study, no significant difference in the abundance of *Enterococcus* was observed between carcinogen-treated rats and those treated with NSS. However, the administration of dried Thua-nao resulted in an increased abundance of this genus in rats treated with DEN and DMH. Furthermore, de Almeida et al. conducted an analysis of the correlation between *E. faecalis* and the development of cancer. They observed that the data only indicated an increase in the concentration of the bacteria but did not provide any information on its specific functional roles in the development of colorectal cancer [52]. In this study, *Alloprevotella* abundance was increased in DEN- and DMH-treated rats. Previous research revealed that *Alloprevotella* was enriched in tumor tissues or feces of individuals with colorectal cancer or adenomas and involved in colorectal carcinogenesis [55,56]. However, administering dried Thua-nao to DEN- and DMH-treated rats resulted in a decrease in the abundance of *Alloprevotella*, which is inconsistent with previous studies [57]. According to Yu et al., a microecological preparation increased the amount of *Alloprevotella* compared to rats that were treated with azoxymethane/dextran sodium sulfate [57]. Moreover, administering dried Thua-nao to DEN- and DMH-treated rats revealed an increase in the amount of *Lactococcus* and *Leuconostoc* in the phylum *Firmicutes*, especially *Lactococcus lactis*, which are known as probiotics. Hosseini et al. found that nisin, a low molecular weight antibacterial peptide that is produced by *L*. *lactis*, has inhibitory effects on cancer cell proliferation, which are linked to the reduced expression of cyclin D1 in the SW480 cancer cell line [58]. This study proposes that dried Thua-nao has the capacity to modulate pathogenic bacteria and beneficial bacteria, hence potentially mitigating the formation of preneoplastic lesions. Nonetheless, clinical investigations about the cancer-preventive efficacy of Thua-nao should be further undertaken.

## 5. Conclusions

These findings indicate the cancer chemopreventive potential of dried Thua-nao in the early stages of colorectal carcinogenesis. Moreover, the inhibitory mechanism of Thua-nao may contribute to the reduction in cell growth and the modulation of gut microbiota. The probable cancer chemopreventive compounds in Thua-nao are isoflavones, including genistein and daidzein. In conclusion, Thua-nao may be a promising food source of beneficial chemopreventive substances for the prevention of cancer or the promotion of health. Moreover, further research is necessary to obtain additional evidence of its effects on chemopreventive potential via clinical studies.

## Figures and Tables

**Table 1:** The contents of total phenolic compounds and isoflavones of dried Thua-nao.

Compounds	Dried Thua-Nao
Spectrophotometry (per 100 g dried Thua-nao)	
Total phenolic compounds (mg GAE)	1180.21 ± 26.63
HPLC (mg per 100 g dried Thua-nao)	
Daidzin	1.68 ± 0.01
Glycitin	ND
Genistin	4.51 ± 0.34
Daidzein	43.45 ± 1.51
Glycitein	6.61 ± 0.02
Genistein	40.71 ± 0.51

Values are expressed as mean ± SD. Gallic acid equivalent (GAE); not detected (ND).

**Table 2:** Effects of dried Thua-nao on general observations.

Group	Treatments	Body Weight (g)		Consumption (Per Rat Per Day)	
		Initial	Final	Diet (g)	Water (mL)
1	DEN + DMH	113 ± 8	453 ± 52	21.3 ± 1.8	29.3 ± 3.1
2	DEN + DMH + TN 100 mg/kg bw	115 ± 10	482 ± 45	23.6 ± 1.7 **	28.7 ± 3.9
3	DEN + DMH + TN 1000 mg/kg bw	114 ± 13	469 ± 21	21.3 ± 1.3	26.5 ± 3.9
4	NSS	115 ± 6	478 ± 35	20.3 ± 1.3	29.7 ± 5.0
5	NSS + TN 1000 mg/kg bw	116 ± 5	543 ± 30 *	24.4 ± 1.6 *	31.9 ± 2.2

The data are expressed as mean ± SD. Diethylnitrosamine (DEN); 1,2-dimethylhydrazine (DMH); normal saline solution (NSS); Thua-nao (TN). * Significantly different compared to the NSS-treated group (*p* < 0.05). ** Significantly different compared to the DEN- and DMH-treated group (*p* < 0.05).

**Table 3:** Effects of dried Thua-nao on some vital organ weights in rats.

Group	Treatments	Liver		Kidney		Spleen	
		Absolute (g)	Relative (%)	Absolute (g)	Relative (%)	Absolute (g)	Relative (%)
1	DEN + DMH	13.80 ± 2.07	3.09 ± 0.69	3.04 ± 1.08	0.70 ± 0.37	0.89 ± 0.27	0.20 ± 0.09
2	DEN + DMH + TN 100 mg/kg bw	14.58 ± 1.76	3.03 ± 0.24	3.07 ± 0.25	0.64 ± 0.04	0.89 ± 0.11	0.18 ± 0.02
3	DEN + DMH + TN 1000 mg/kg bw	13.66 ± 1.42	2.92 ± 0.30	2.99 ± 0.16	0.64 ± 0.03	0.86 ± 0.08	0.18 ± 0.02
4	NSS	12.78 ± 1.32	2.67 ± 0.19	2.70 ± 0.27	0.57 ± 0.04	0.77 ± 0.06	0.16 ± 0.02
5	NSS + TN 1000 mg/kg bw	16.36 ± 1.54 *	3.01 ± 0.22	3.07 ± 0.29	0.56 ± 0.03	0.70 ± 0.10	0.13 ± 0.02

The data are presented as mean ± SD. Diethylnitrosamine (DEN); 1,2-dimethylhydrazine (DMH); normal saline solution (NSS); Thua-nao (TN). * Significantly different compared to the NSS-treated group (*p* < 0.05).

**Table 4:** Effects of dried Thua-nao on preneoplastic lesion formation in DEN- and DMH-treated rats.

Group	Treatments	Liver		Colon	
		Number of GST-P-Positive Foci/Liver Area (cm^2^)	Area of GST-P-Positive Foci (mm^2^)/Liver Area (cm^2^)	Total Aberrant Crypt Foci/Rat	Aberrant Crypt/Focus
1	DEN + DMH	20.8 ± 7.4 *	1.12 ± 0.51 *	148.9 ± 52.2 *	4.90 ± 0.44 *
2	DEN + DMH + TN 100 mg/kg bw	19.1 ± 5.7	0.97 ± 0.20	126.1 ± 57.7	5.11 ± 0.43
3	DEN + DMH + TN 1000 mg/kg bw	14.9 ± 4.8	0.69 ± 0.60	87.8 ± 43.5 **	4.51 ± 0.44
4	NSS	0.0 ± 0.0	0.00 ± 0.00	0.0 ± 0.0	0.00 ± 0.00
5	NSS + TN 1000 mg/kg bw	0.0 ± 0.0	0.00 ± 0.00	0.0 ± 0.0	0.00 ± 0.00

The data are shown as mean ± SD. Diethylnitrosamine (DEN); 1,2-dimethylhydrazine (DMH); normal saline solution (NSS); Thua-nao (TN). * Significantly different compared to the NSS-treated group (*p* < 0.05). ** Significantly different compared to the DEN- and DMH-treated group (*p* < 0.05).

## Data Availability

The original contributions presented in the study are included in the article/Supplementary Material; further inquiries can be directed to the corresponding author.

## Supplementary Materials

The following supporting information can be downloaded at: https://www.mdpi.com/article/10.3390/nu16203506/s1, Table S1: The nutritional composition of commercial basal diets; Table S2: Differential abundance analysis results (Genus levels).
