# Supplementary material for: Thai Fermented Soybean (Thua-Nao) Prevents Early Stages of Colorectal Carcinogenesis Induced by Diethylnitrosamine and 1,2-Dimethylhydrazine Through Modulations of Cell Proliferation and Gut Microbiota in Rats
Source: Nutrients. 2024 Oct 16;16(20):3506. doi: 10.3390/nu16203506 (PMC11510544; doi:10.3390/nu16203506)
Supplement: Supplementary file 1 [file nutrients-16-03506-s001.zip › nutrients-3227470-supplementary.pdf]

Supplemental Material

Thai Fermented Soybean (Thua-Nao) Prevents Early Stages of Colorectal Carcinogenesis Induced by Diethylnitrosamine and 1,2-Dimethylhydrazine through Modulations of Cell Proliferation and Gut Microbiota in Rats

Sirinya Taya [1,*], Sivamoke Dissook [2], Jetsada Ruangsuriya [1,2], Supachai Yodkeeree [2], Kongsak Boonyapranai [3], Teera Chewonarin [2] and Rawiwan Wongpoomchai [2]

[1] Functional Food Research Unit, Multidisciplinary Research Institute, Chiang Mai University, Chiang Mai 50200, Thailand
[2] Department of Biochemistry, Faculty of Medicine, Chiang Mai University, Chiang Mai 50200, Thailand
[3] Research Institute for Health Sciences, Chiang Mai University, Chiang Mai 50200, Thailand
* Correspondence: sirinya.t@cmu.ac.th

Outline

Table S1..........3

Table S2..........4

Table S1. *The nutritional composition of commercial basal diets.*

**Nutritional composition of C.P. mice feed (082G)**		
Moisture	(Max)	12 %
Crude protein	(Min)	24 %
Fat	(Min)	4.5 %
Fiber	(Max)	5 %
Metabolizable energy	(swing) Kcal/kg.	3,040
Calcium		1.0 %
Phosphorus (available)		0.9 %
Sodium		0.20 %
Potassium		1.17 %
Magnesium		0.23 %
Manganese	p.p.m.	171
Copper	p.p.m.	22
Zinc	p.p.m.	100
Iron	p.p.m.	180
Cobalt	p.p.m.	1.82
Potassium Iodide	p.p.m.	1
Selenium	p.p.m.	0.1
**Vitamins**		
A	i.u. / kg.	20,000
D	i.u. / kg.	4,000
E	mg / kg.	100
K	mg / kg.	5
B1	mg / kg.	20
B2	mg / kg.	20
B6	mg / kg.	20
B12	mg / kg.	0.036
Niacin	mg / kg.	100
Folic Acid	mg / kg.	6
Biotin	mg / kg.	0.4
Pantothenic Acid	mg / kg.	60
Choline Chloride	mg / kg.	1,500

Table S2. *Differential-abundance-analysis-results (Genus levels).*

Differential-abundance-analysis-results (Genus levels)	kw.ep	kw.eBH	glm.ep	glm.eBH
d__Bacteria;p__Firmicutes;c__Clostridia;o__Lachnospirales;f__Defluviitaleaceae;g__Defluviitaleaceae_UCG-011	0.02022376	0.24908825	0.00017788	0.00788798
d__Bacteria;p__Firmicutes;c__Bacilli;o__Lactobacillales;f__Streptococcaceae;g__Lactococcus	0.00687121	0.21813011	0.00045905	0.0119785
d__Bacteria;p__Actinobacteriota;c__Coriobacteriia;o__Coriobacteriales;f__Eggerthellaceae;g__Adlercreutzia	0.01202502	0.22331365	0.00131466	0.02050219
d__Bacteria;p__Firmicutes;c__Bacilli;o__Lactobacillales;f__Leuconostocaceae;g__Leuconostoc	0.00940905	0.22130299	0.00154503	0.02347463
d__Bacteria;p__Firmicutes;c__Bacilli;o__Bacillales;f__Bacillaceae;g__Bacillus	0.00873869	0.21926716	0.00196285	0.02694311
d__Bacteria;p__Actinobacteriota;c__Coriobacteriia;o__Coriobacteriales;f__Atopobiaceae;g__uncultured	0.01699136	0.2410467	0.00384836	0.03505061
d__Bacteria;p__Firmicutes;c__Clostridia;o__Oscillospirales;f__Ruminococcaceae;g__Ruminococcaceae	0.01329412	0.22094483	0.00794673	0.06048882
d__Bacteria;p__Actinobacteriota;c__Coriobacteriia;o__Coriobacteriales;f__Eggerthellaceae;g__Enterorhabdus	0.03582696	0.27357018	0.00874144	0.08249121
d__Bacteria;p__Firmicutes;c__Clostridia;o__Oscillospirales;f__Ruminococcaceae;g__Incertae_Sedis	0.02586325	0.25896576	0.00971971	0.05701839
d__Bacteria;p__Bacteroidota;c__Bacteroidia;o__Bacteroidales;f__Prevotellaceae;g__Alloprevotella	0.02487277	0.24253985	0.01378238	0.08973747
d__Bacteria;p__Firmicutes;c__Clostridia;o__Oscillospirales;f__Ruminococcaceae;g__UBA1819	0.04471301	0.30698736	0.01641923	0.07869433
d__Bacteria;p__Firmicutes;c__Clostridia;o__Christensenellales;f__Christensenellaceae;g__Christensenella	0.04377061	0.28037628	0.01866259	0.09898694
d__Bacteria;p__Firmicutes;c__Clostridia;o__Oscillospirales;f__Oscillospiraceae;g__Oscillibacter	0.03406534	0.2719895	0.02570995	0.10313641
d__Bacteria;p__Firmicutes;c__Clostridia;o__uncultured;f__uncultured;g__uncultured	0.05648572	0.33167981	0.02632065	0.16204964
d__Bacteria;p__Firmicutes;c__Clostridia;o__Peptostreptococcales-Tissierellales;f__Anaerovoracaceae;g__Family_XIII_AD3011_group	0.03614316	0.28964137	0.02639281	0.14216044
d__Bacteria;p__Bacteroidota;c__Bacteroidia;o__Bacteroidales;f__Bacteroidaceae;g__Bacteroides	0.06587439	0.34991206	0.02829297	0.16331213
d__Bacteria;p__Firmicutes;c__Clostridia;o__Lachnospirales;f__Lachnospiraceae;__	0.05168568	0.3116481	0.0305566	0.17009894
d__Bacteria;p__Patescibacteria;c__Saccharimonadia;o__Saccharimonadales;f__Saccharimonadaceae;g__Candidatus_Saccharimonas	0.00364452	0.21451718	0.03447897	0.17657646
d__Bacteria;p__Firmicutes;c__Clostridia;o__Peptostreptococcales-Tissierellales;f__Anaerovoracaceae;g__[Eubacterium]_nodatum_group	0.07074557	0.34138755	0.04614546	0.19428072
d__Bacteria;p__Proteobacteria;c__Alphaproteobacteria;o__Rhodospirillales;f__uncultured;g__uncultured	0.05630118	0.32513804	0.05488332	0.2022375
d__Bacteria;p__Firmicutes;c__Bacilli;o__Lactobacillales;f__Enterococcaceae;g__Enterococcus	0.01491723	0.22598313	0.05611587	0.20902885
d__Bacteria;p__Bacteroidota;c__Bacteroidia;o__Bacteroidales;f__Rikenellaceae;g__Alistipes	0.14717802	0.44711707	0.0576166	0.23041276
d__Bacteria;p__Firmicutes;c__Clostridia;o__Oscillospirales;f__UCG-010;g__UCG-010	0.31469953	0.59872795	0.05837927	0.21578873
d__Bacteria;p__Firmicutes;c__Bacilli;o__Staphylococcales;f__Staphylococcaceae;g__Staphylococcus	0.0655821	0.34482112	0.06244016	0.22072193
d__Bacteria;p__Firmicutes;c__Bacilli;o__Erysipelotrichales;f__Erysipelotrichaceae;g__Erysipelotrichaceae	0.17261748	0.46307183	0.06500175	0.21691992
d__Bacteria;p__Bacteroidota;c__Bacteroidia;o__Bacteroidales;f__Muribaculaceae;g__Muribaculum	0.14584159	0.42097408	0.07382233	0.21962129
d__Bacteria;p__Desulfobacterota;c__Desulfovibrionia;o__Desulfovibrionales;f__Desulfovibrionaceae;g__Bilophila	0.16456432	0.45869171	0.07786744	0.25356039
d__Bacteria;p__Firmicutes;c__Bacilli;o__Lactobacillales;f__Streptococcaceae;g__Streptococcus	0.10448465	0.390197	0.08551961	0.27260475
d__Bacteria;p__Firmicutes;c__Clostridia;o__Peptostreptococcales-Tissierellales;f__Anaerovoracaceae;g__Anaerovorax	0.12203243	0.41124022	0.08756777	0.26559261
d__Bacteria;p__Firmicutes;c__Clostridia;o__Clostridia;f__Hungateiclostridiaceae;g__Ruminiclostridium	0.27709345	0.57086413	0.09025042	0.27868462
d__Bacteria;p__Firmicutes;c__Clostridia;o__Lachnospirales;f__Lachnospiraceae;g__Lachnospiraceae_NK4B4_group	0.13046826	0.41091397	0.0914598	0.24589097

d__Bacteria;p__Firmicutes;c__Bacilli;o__Erysipelotrichales;f__Erysipelotrichaceae;g__Allobaculum	0.2447506	0.4882791	0.09453829	0.2411139
d__Bacteria;p__Firmicutes;c__Clostridia;o__Oscillospirales;f__Oscillospiraceae;g__UCG-005	0.26398409	0.5636611	0.09841203	0.29731264
d__Bacteria;p__Bacteroidota;c__Bacteroidia;o__Bacteroidales;f__Muribaculaceae;g__Muribaculaceae	0.20803329	0.50885068	0.10255376	0.30137466
d__Bacteria;p__Firmicutes;c__Bacilli;o__Acholeplasmatales;f__Acholeplasmataceae;g__Anaeroplasma	0.20499215	0.45871751	0.10692874	0.23699718
d__Bacteria;p__Firmicutes;c__Bacilli;o__Erysipelotrichales;f__Erysipelatoclostridiaceae;g__Erysipelotrichaceae_UCG-003	0.22001022	0.49981607	0.10912342	0.28828413
d__Bacteria;p__Firmicutes;c__Clostridia;o__Lachnospirales;f__Lachnospiraceae;g__[Bacteroides]_pectinophilus_group	0.14176664	0.41381728	0.11363532	0.24811207
d__Bacteria;p__Firmicutes;c__Clostridia;o__Oscillospirales;f__Ruminococcaceae;g__Pygmaiobacter	0.21480845	0.49698377	0.11520982	0.28524287
d__Bacteria;p__Firmicutes;c__Bacilli;o__Erysipelotrichales;f__Erysipelotrichaceae;g__Turicibacter	0.13505989	0.41626537	0.11751941	0.31619648
d__Bacteria;p__Firmicutes;c__Bacilli;o__Izemoplasmatales;f__Izemoplasmatales;g__Izemoplasmatales	0.14564124	0.42588877	0.13492421	0.31962308
d__Bacteria;p__Firmicutes;c__Clostridia;o__Christensenellales;f__Christensenellaceae;g__Christensenellaceae	0.15518754	0.42533574	0.13559068	0.28618438
d__Bacteria;p__Actinobacteriota;c__Coriobacteriia;o__Coriobacteriales;f__Eggerthellaceae;g__uncultured	0.144392	0.44727012	0.14210438	0.35187518
d__Bacteria;p__Firmicutes;c__Bacilli;o__Staphylococcales;f__Gemellaceae;g__Gemella	0.12366034	0.39321888	0.14601609	0.27048628
d__Bacteria;p__Firmicutes;c__Clostridia;o__Oscillospirales;f__Ethanoligenenaceae;g__Acetanaerobacterium	0.12600539	0.3950887	0.15556634	0.27378864
d__Bacteria;p__Firmicutes;c__Bacilli;o__RF39;f__RF39;g__RF39	0.12508794	0.42219638	0.15662628	0.37387873
d__Bacteria;p__Firmicutes;c__Clostridia;o__Lachnospirales;f__Lachnospiraceae;g__[Eubacterium]_xylanophilum_group	0.07826632	0.36365513	0.16115356	0.36720014
d__Bacteria;p__Firmicutes;c__Clostridia;o__Oscillospirales;f__Ruminococcaceae;g__Fournierella	0.16957871	0.45550661	0.16375331	0.31869236
d__Bacteria;p__Actinobacteriota;c__Coriobacteriia;o__Coriobacteriales;f__Eggerthellaceae;__	0.19735763	0.49914684	0.16566345	0.37479339
d__Bacteria;p__Firmicutes;c__Clostridia;o__Lachnospirales;f__Lachnospiraceae;g__Roseburia	0.24257433	0.50455415	0.16574833	0.33333684
d__Bacteria;p__Firmicutes;c__Clostridia;o__Lachnospirales;f__Lachnospiraceae;g__Lachnospiraceae_NK4A136_group	0.50723012	0.73331832	0.17026934	0.38235835
d__Bacteria;p__Firmicutes;c__Clostridia;o__Lachnospirales;f__Lachnospiraceae;g__uncultured	0.16142935	0.46007049	0.17200338	0.38313512
d__Bacteria;p__Proteobacteria;c__Gammaproteobacteria;o__Burkholderiales;f__Sutterellaceae;g__Parasutterella	0.10279537	0.38308828	0.1815844	0.3980239
d__Bacteria;p__Firmicutes;c__Clostridia;o__Lachnospirales;f__Lachnospiraceae;g__Dorea	0.23231482	0.51610837	0.18854874	0.3776715
d__Bacteria;p__Firmicutes;c__Bacilli;o__Lactobacillales;f__Leuconostocaceae;g__Weissella	0.12259023	0.40665511	0.19203409	0.34849945
d__Bacteria;p__Firmicutes;c__Bacilli;o__Erysipelotrichales;f__Erysipelotrichaceae;g__[Clostridium]_innocuum_group	0.22834843	0.47256585	0.19795128	0.32111697
d__Bacteria;p__Firmicutes;c__Clostridia;o__Lachnospirales;f__Lachnospiraceae;g__Lachnoclostridium	0.14945582	0.44061889	0.20119717	0.4095496
d__Bacteria;p__Firmicutes;c__Clostridia;o__Lachnospirales;f__Lachnospiraceae;g__Sellimonas	0.19656726	0.48410386	0.20999296	0.37988363
d__Bacteria;p__Cyanobacteria;c__Vampirivibrionia;o__Gastranaerophilales;f__Gastranaerophilales;g__Gastranaerophilales	0.36091069	0.6309725	0.21007834	0.42994326
d__Bacteria;p__Actinobacteriota;c__Coriobacteriia;o__Coriobacteriales;f__Eggerthellaceae;g__Enteroscipio	0.40376931	0.63656671	0.22155589	0.40614818
d__Bacteria;p__Firmicutes;c__Clostridia;o__Lachnospirales;f__Lachnospiraceae;g__GCA-900066575	0.24537088	0.51087719	0.22633165	0.40762736
d__Bacteria;p__Firmicutes;c__Clostridia;o__Oscillospirales;f__Oscillospiraceae;g__Papillibacter	0.26140242	0.52387954	0.23728268	0.39579852
Unassigned;__;__;__;__;__	0.32170516	0.57015873	0.24029083	0.3908287
d__Bacteria;p__Firmicutes;c__Clostridia;o__Oscillospirales;__;__	0.23877959	0.51897334	0.2447086	0.42177012

d__Bacteria;p__Firmicutes;c__Bacilli;o__Lactobacillales;f__Lactobacillaceae;g__Pediococcus	0.31679288	0.57704176	0.2495475	0.41715637
d__Bacteria;p__Firmicutes;c__Clostridia;o__Oscillospirales;f__Oscillospiraceae;g__NK4A214_group	0.30557376	0.56802949	0.25199188	0.42760896
d__Bacteria;p__Firmicutes;c__Clostridia;o__Lachnospirales;f__Lachnospiraceae;g__Moryella	0.32766224	0.58091274	0.25479387	0.43147641
d__Bacteria;p__Firmicutes;c__Clostridia;o__Oscillospirales;f__Butyricicoccaceae;g__UCG-009	0.26293178	0.55541127	0.27666387	0.48287552
d__Bacteria;p__Proteobacteria;c__Gammaproteobacteria;o__Enterobacterales;f__Enterobacteriaceae;g__Escherichia-Shigella	0.31960254	0.57258097	0.27683416	0.44652422
d__Bacteria;p__Firmicutes;c__Clostridia;o__Peptostreptococcales-Tissierellales;f__Anaerovoracaceae;__	0.35362829	0.59654012	0.28412619	0.48239921
d__Bacteria;p__Firmicutes;c__Clostridia;o__Peptococcales;f__Peptococcaceae;g__uncultured	0.36113252	0.6451724	0.28692207	0.49615726
d__Bacteria;p__Firmicutes;c__Bacilli;o__Erysipelotrichales;f__Erysipelotrichaceae;g__Dielma	0.34231352	0.5734789	0.29305175	0.44194378
d__Bacteria;p__Desulfobacterota;c__Desulfovibrionia;o__Desulfovibrionales;f__Desulfovibrionaceae;g__uncultured	0.38915223	0.62625153	0.29823206	0.46860665
d__Bacteria;p__Firmicutes;c__Clostridia;o__Oscillospirales;f__[Clostridium]_methylpentosum_group;g__[Clostridium]_methylpentosum_group	0.36265796	0.61341276	0.30671663	0.46813786
d__Bacteria;p__Firmicutes;c__Clostridia;o__Christensenellales;f__Christensenellaceae;__	0.43338876	0.64084593	0.31919028	0.46334099
d__Bacteria;p__Firmicutes;c__Incertae_Sedis;o__DTU014;f__DTU014;g__DTU014	0.31698893	0.58900548	0.32029399	0.47710933
d__Bacteria;p__Firmicutes;c__Bacilli;o__Erysipelotrichales;f__Erysipelotrichaceae;g__Dubosiella	0.3772202	0.61943764	0.32312275	0.5032755
d__Bacteria;p__Firmicutes;c__Clostridia;o__Lachnospirales;f__Lachnospiraceae;g__Coprococcus	0.40984364	0.6364873	0.32720053	0.50156812
d__Bacteria;p__Firmicutes;c__Bacilli;o__Erysipelotrichales;f__Erysipelatoclostridiaceae;g__Candidatus_Stoquefichus	0.42516638	0.64044247	0.33794953	0.50676309
d__Bacteria;p__Firmicutes;c__Clostridia;o__Oscillospirales;f__Oscillospiraceae;g__Colidextribacter	0.4407054	0.69731694	0.33859492	0.54707603
d__Bacteria;p__Firmicutes;c__Bacilli;o__Erysipelotrichales;f__Erysipelotrichaceae;g__Holdemania	0.45337508	0.68485358	0.34340591	0.53302191
d__Bacteria;p__Firmicutes;c__Clostridia;o__Oscillospirales;f__Ruminococcaceae;g__uncultured	0.80310783	0.89596627	0.35438024	0.57401634
d__Bacteria;p__Firmicutes;c__Clostridia;o__Oscillospirales;f__Ruminococcaceae;g__Candidatus_Soleaferrea	0.47143835	0.71589375	0.36245598	0.56543103
d__Bacteria;p__Firmicutes;c__Bacilli;o__Staphylococcales;f__Staphylococcaceae;g__Macrococcus	0.43487447	0.63472991	0.36308946	0.52981161
d__Bacteria;p__Firmicutes;c__Clostridia;o__Oscillospirales;f__Ruminococcaceae;g__Negativibacillus	0.41169064	0.63416038	0.37098591	0.52794689
d__Bacteria;p__Firmicutes;c__Clostridia;o__Lachnospirales;f__Lachnospiraceae;g__[Eubacterium]_oxidoreducens_group	0.4631464	0.69265134	0.37242256	0.55015929
d__Bacteria;p__Bacteroidota;c__Bacteroidia;o__Bacteroidales;f__Tannerellaceae;g__Candidatus_Vestibaculum	0.4553906	0.68961577	0.3737412	0.56864495
d__Bacteria;p__Firmicutes;c__Clostridia;o__Oscillospirales;f__Ruminococcaceae;g__Angelakisella	0.42693246	0.64229143	0.37681423	0.54318086
d__Bacteria;p__Bacteroidota;c__Bacteroidia;o__Bacteroidales;f__Tannerellaceae;g__Tannerellaceae	0.4726874	0.6993079	0.37795246	0.5558265
d__Bacteria;p__Actinobacteriota;c__Coriobacteriia;o__Coriobacteriales;f__Eggerthellaceae;g__Gordonibacter	0.45477285	0.69958767	0.38292892	0.58561442
d__Bacteria;p__Firmicutes;c__Clostridia;o__Christensenellales;f__Christensenellaceae;g__uncultured	0.47565241	0.69367126	0.38325395	0.5705694
d__Bacteria;p__Firmicutes;c__Clostridia;o__Clostridia_vadinBB60_group;f__Clostridia_vadinBB60_group;g__Clostridia_vadinBB60_group	0.42900295	0.67191165	0.3872963	0.55510028
d__Bacteria;p__Firmicutes;c__Clostridia;o__Lachnospirales;f__Lachnospiraceae;g__Tyzzerella	0.49992743	0.72314346	0.38806705	0.57209691
d__Bacteria;p__Firmicutes;c__Bacilli;o__Lactobacillales;f__Carnobacteriaceae;g__Carnobacterium	0.48150679	0.69950178	0.38902298	0.58126897
d__Bacteria;p__Bacteroidota;c__Bacteroidia;o__Bacteroidales;f__Marinifilaceae;g__Butyricimonas	0.4707218	0.67610956	0.39229709	0.54114475
d__Bacteria;p__Bacteroidota;c__Bacteroidia;o__Bacteroidales;f__Prevotellaceae;g__Prevotella	0.39751887	0.62122588	0.39550928	0.52992827
d__Bacteria;p__Firmicutes;c__Bacilli;o__Erysipelotrichales;f__Erysipelatoclostridiaceae;g__Erysipelatoclostridium	0.48991951	0.72054718	0.39566746	0.59896515

d__Bacteria;p__Firmicutes;c__Clostridia;o__Clostridiales;f__uncultured;g__uncultured	0.41343443	0.62838016	0.40145791	0.54941059
d__Bacteria;p__Firmicutes;c__Clostridia;o__Monoglobales;f__Monoglobaceae;g__Monoglobus	0.29635069	0.58454697	0.40630924	0.60973556
d__Bacteria;p__Firmicutes;c__Clostridia;o__Lachnospirales;f__Lachnospiraceae;g__[Eubacterium]_fissicatena_group	0.47719261	0.69246314	0.41204462	0.57782527
d__Bacteria;p__Firmicutes;c__Clostridia;o__Oscillospirales;f__Ruminococcaceae;__	0.73159494	0.85235072	0.41418883	0.61986547
d__Bacteria;p__Firmicutes;c__Clostridia;o__Lachnospirales;f__Lachnospiraceae;g__[Ruminococcus]_torques_group	0.49104282	0.71209048	0.41571519	0.58784961
d__Bacteria;__;__;__;__;__	0.41944425	0.63920168	0.41783452	0.57501868
d__Bacteria;p__Firmicutes;c__Clostridia;o__Oscillospirales;f__[Eubacterium]_coprostanoligenes_group;g__[Eubacterium]_coprostanoligenes_group	0.51973027	0.7443808	0.42008339	0.61336761
d__Bacteria;p__Firmicutes;c__Clostridia;o__Oscillospirales;f__Butyricicoccaceae;g__Butyricicoccus	0.49570388	0.70316993	0.42176203	0.58885728
d__Bacteria;p__Firmicutes;c__Clostridia;o__Lachnospirales;f__Lachnospiraceae;g__Lachnospiraceae_FCS020_group	0.4531036	0.68459611	0.42525444	0.60047457
d__Bacteria;p__Actinobacteriota;c__Coriobacteriia;o__Coriobacteriales;f__Atopobiaceae;g__Coriobacteriaceae_UCG-002	0.58120619	0.76064214	0.43331671	0.59918367
d__Bacteria;p__Firmicutes;c__Bacilli;o__Erysipelotrichales;f__Erysipelotrichaceae;g__uncultured	0.59948271	0.78732673	0.43351366	0.63130938
d__Bacteria;p__Firmicutes;c__Clostridia;o__Oscillospirales;f__Ruminococcaceae;g__Ruminococcus	0.4561959	0.71214672	0.43445593	0.63178032
d__Bacteria;p__Firmicutes;c__Clostridia;o__Oscillospirales;f__Oscillospiraceae;g__UCG-002	0.53254355	0.70550982	0.43453448	0.57754883
d__Bacteria;p__Firmicutes;c__Clostridia;o__Lachnospirales;f__Lachnospiraceae;g__[Eubacterium]_ventriosum_group	0.49575321	0.70965105	0.43823049	0.61037931
d__Bacteria;p__Firmicutes;c__Clostridia;o__Lachnospirales;f__Lachnospiraceae;g__[Eubacterium]_ruminantium_group	0.47831131	0.67644901	0.43876825	0.59517749
d__Bacteria;p__Firmicutes;c__Clostridia;o__Peptostreptococcales-Tissierellales;f__Anaerovoracaceae;g__uncultured	0.45908359	0.67193968	0.44256987	0.60310175
d__Bacteria;p__Actinobacteriota;c__Actinobacteria;o__Corynebacteriales;f__Corynebacteriaceae;g__Corynebacterium	0.46459558	0.69843893	0.44426001	0.61077408
d__Bacteria;p__Bacteroidota;c__Bacteroidia;o__Bacteroidales;f__Prevotellaceae;g__Prevotellaceae_UCG-001	0.58318805	0.77297021	0.44654526	0.63996561
d__Bacteria;p__Firmicutes;c__Clostridia;o__Oscillospirales;f__Ruminococcaceae;g__Paludicola	0.46019633	0.67967225	0.44700051	0.60241586
d__Bacteria;p__Firmicutes;c__Clostridia;o__Oscillospirales;f__Oscillospirales;g__Hydrogenoanaerobacterium	0.34836708	0.62158804	0.45292325	0.63750584
d__Bacteria;p__Actinobacteriota;c__Coriobacteriia;o__Coriobacteriales;f__Eggerthellaceae;g__Eggerthella	0.49213162	0.70234597	0.45368701	0.6231244
d__Bacteria;p__Firmicutes;c__Clostridia;o__Lachnospirales;f__Lachnospiraceae;g__Lachnospiraceae_ND3007_group	0.499691	0.69353594	0.45405329	0.60563058
d__Bacteria;p__Firmicutes;c__Bacilli;o__Staphylococcales;f__Staphylococcaceae;g__Jeotgalicoccus	0.47434224	0.68592597	0.46230205	0.60416904
d__Bacteria;p__Firmicutes;c__Clostridia;o__Oscillospirales;f__Oscillospiraceae;g__Flavonifractor	0.53846636	0.72777859	0.46413119	0.61535393
d__Bacteria;p__Actinobacteriota;c__Actinobacteria;o__Bifidobacteriales;f__Bifidobacteriaceae;g__Bifidobacterium	0.42878387	0.6650537	0.46604753	0.62634872
d__Bacteria;p__Firmicutes;c__Clostridia;o__Oscillospirales;f__Ruminococcaceae;g__Anaerotruncus	0.47281968	0.68945468	0.46671606	0.60529084
d__Bacteria;p__Actinobacteriota;c__Actinobacteria;o__Actinomycetales;f__Actinomycetaceae;g__Actinomyces	0.60489803	0.7878616	0.46756357	0.62944817
d__Bacteria;p__Proteobacteria;c__Gammaproteobacteria;o__Enterobacterales;f__Morganellaceae;g__Proteus	0.42014684	0.66937015	0.46795405	0.63354082
d__Bacteria;p__Actinobacteriota;c__Actinobacteria;o__Micrococcales;f__Micrococcaceae;g__Rothia	0.5464778	0.72474092	0.46886146	0.61125619
d__Bacteria;p__Firmicutes;c__Clostridia;o__Oscillospirales;f__Ruminococcaceae;g__Harryflintia	0.55301671	0.74419719	0.4691396	0.62266019
d__Bacteria;p__Firmicutes;c__Bacilli;o__Erysipelotrichales;f__Erysipelotrichaceae;g__Faecalibaculum	0.53233108	0.7277862	0.47171933	0.62159061
d__Bacteria;p__Firmicutes;c__Clostridia;o__Peptostreptococcales-Tissierellales;f__Peptostreptococcaceae;g__Romboutsia	0.60233178	0.78712994	0.47637778	0.65996754
d__Bacteria;p__Proteobacteria;c__Gammaproteobacteria;o__Enterobacterales;f__Enterobacteriaceae;__	0.50057117	0.70355214	0.48360626	0.6318014

d__Bacteria;p__Firmicutes;c__Clostridia;__;__;__	0.51324008	0.74431213	0.48739379	0.669114
d__Bacteria;p__Proteobacteria;c__Alphaproteobacteria;o__Rhizobiales;f__Rhizobiaceae;g__Pseudochrobactrum	0.57031044	0.75366935	0.4961318	0.66704866
d__Bacteria;p__Actinobacteriota;c__Actinobacteria;o__Micrococcales;f__Microbacteriaceae;g__Microbacterium	0.5298903	0.71376962	0.53370752	0.6705074
d__Bacteria;p__Bacteroidota;c__Bacteroidia;o__Bacteroidales;f__Tannerellaceae;g__Parabacteroides	0.60270488	0.78979254	0.53885345	0.71168975
d__Bacteria;p__Firmicutes;c__Clostridia;o__Clostridiales;f__Clostridiaceae;g__Clostridium_sensu_stricto_1	0.53677876	0.73779738	0.54529797	0.68213875
d__Bacteria;p__Firmicutes;c__Bacilli;o__Lactobacillales;f__Lactobacillaceae;g__Lactobacillus	0.64609289	0.8120524	0.54930792	0.70853424
d__Bacteria;p__Firmicutes;c__Clostridia;o__Peptostreptococcales-Tissierellales;f__Anaerovoracaceae;g__[Eubacterium]_brachy_group	0.60533079	0.78006516	0.55156584	0.67823934
d__Bacteria;p__Firmicutes;c__Bacilli;o__Lactobacillales;f__Carnobacteriaceae;g__Atopostipes	0.67071578	0.80732911	0.55775941	0.68100981
d__Bacteria;p__Firmicutes;c__Clostridia;o__Christensenellales;f__Christensenellaceae;g__Christensenellaceae_R-7_group	0.5181283	0.74371799	0.56867644	0.71724303
d__Bacteria;p__Firmicutes;c__Clostridia;o__Lachnospirales;f__Lachnospiraceae;g__Lachnospiraceae_NC2004_group	0.64189095	0.79892194	0.58165849	0.71184005
d__Bacteria;p__Firmicutes;c__Clostridia;o__Oscillospirales;f__Ruminococcaceae;g__[Eubacterium]_siraeum_group	0.64175121	0.79912547	0.58743276	0.71813917
d__Bacteria;p__Bacteroidota;c__Bacteroidia;o__Bacteroidales;f__Rikenellaceae;g__Rikenellaceae	0.65605377	0.81473791	0.59063269	0.72448809
d__Bacteria;p__Firmicutes;c__Clostridia;o__Clostridia_UCG-014;f__Clostridia_UCG-014;g__Clostridia_UCG-014	0.71795419	0.84349704	0.63161781	0.75782338
d__Bacteria;p__Firmicutes;c__Clostridia;o__Oscillospirales;f__Oscillospiraceae;g__Oscillospira	0.69360334	0.83005125	0.63846714	0.75999329
d__Bacteria;p__Firmicutes;c__Clostridia;o__Oscillospirales;f__uncultured;g__uncultured	0.64411466	0.81024918	0.64420343	0.76479019
d__Bacteria;p__Firmicutes;c__Bacilli;o__Erysipelotrichales;f__Erysipelotrichaceae;g__Faecalitalea	0.6760739	0.81817081	0.64460624	0.7575899
d__Bacteria;p__Firmicutes;c__Clostridia;o__Peptostreptococcales-Tissierellales;__;__	0.96494606	0.97979727	0.65362954	0.76767958
d__Bacteria;p__Firmicutes;c__Clostridia;o__Lachnospirales;f__Lachnospiraceae;g__Marvinbryantia	0.76810278	0.87456706	0.65552855	0.77689745
d__Bacteria;p__Firmicutes;c__Clostridia;o__Oscillospirales;f__Oscillospiraceae;g__uncultured	0.57851878	0.76001115	0.66525181	0.77608507
d__Bacteria;p__Firmicutes;c__Clostridia;o__Oscillospirales;f__Oscillospiraceae;g__UCG-007	0.71791826	0.85479781	0.6664402	0.78747634
d__Bacteria;p__Firmicutes;c__Clostridia;o__Eubacteriales;f__Anaerofustaceae;g__Anaerofustis	0.65617126	0.81591952	0.69916744	0.8096473
d__Bacteria;p__Firmicutes;c__Clostridia;o__Oscillospirales;f__Oscillospiraceae;__	0.63777338	0.8078745	0.7428948	0.83123838
d__Bacteria;p__Firmicutes;c__Clostridia;o__Lachnospirales;f__Lachnospiraceae;g__Frisingicoccus	0.96168657	0.97993179	0.76294838	0.85068652
d__Bacteria;p__Firmicutes;c__Clostridia;o__Lachnospirales;f__Lachnospiraceae;g__Blautia	0.60160985	0.79380861	0.78582717	0.86114101
d__Bacteria;p__Verrucomicrobiota;c__Verrucomicrobiae;o__Verrucomicrobiales;f__Akkermansiaceae;g__Akkermansia	0.98455068	0.98944643	0.94015538	0.95895547
d__Bacteria;p__Bacteroidota;c__Bacteroidia;o__Bacteroidales;f__Rikenellaceae;g__Rikenella	0.90921971	0.95727702	0.96134172	0.97093636
